# Investigation of the Flame-Retardant and Mechanical Properties of Bamboo Fiber-Reinforced Polypropylene Composites with Melamine Pyrophosphate and Aluminum Hypophosphite Addition

**DOI:** 10.3390/ma13020479

**Published:** 2020-01-19

**Authors:** Lu Fang, Xizhen Lu, Jian Zeng, Yingyi Chen, Qiheng Tang

**Affiliations:** 1 Co-Innovation Center of Efficient Processing and Utilization of Forest Resources, Nanjing Forestry University, Nanjing 210037, China; fanglu@njfu.edu.cn; 2 College of Furnishings and Industrial Design, Nanjing Forestry University, Nanjing 210037, China; luxizhenxx@126.com (X.L.); zengj1996222@163.com (J.Z.); lemon1119yiyi@163.com (Y.C.); 3 Research Institute of Wood Industry, Chinese Academy of Forestry, Beijing 100091, China

**Keywords:** bamboo fiber/polypropylene composites, flame retardancy, melamine pyrophosphate, aluminum hypophosphite

## Abstract

To improve the flame-retardant performance of bamboo fiber (BF) reinforced polypropylene (PP) composites, melamine pyrophosphate (MPP) and aluminum hypophosphite (AP) at a constant mass ratio of 2:1 were added. The influence of the MPP/AP mass fraction on the mechanical and flame-retardant properties of the BF reinforced PP composites were evaluated by mechanical testing, limiting oxygen index (LOI) and cone calorimetry. Mechanical tests demonstrate that tensile properties of BF/PP decreased with the increase of MPP/AP mass fraction, while flexural properties of composites exhibited very different tendencies. Both flexural strength and modulus increased slightly with the addition of MPP/AP at first, and then decreased significantly after a relatively high content of MPP/AP was loaded. This was due to the poor interfacial compatibility between PP and MPP/AP. The flame retardancy of BF/PP composites has been greatly improved. When 30% MPP/AP was loaded into the composites, the LOI increased to 27.2%, which was 42.4% higher than that of the composite without flame retardant addition. Cone calorimetry results indicated that MPP/AP worked in both gas and condensed phases during the combustion process. Peak heat release rate, total smoke production and mass loss of the composites were significantly reduced because of the addition of MPP/AP.

## 1. Introduction

The substantial attention to the global environment and ever-growing awareness of the renewable need for green resources has motivated a lot of scientific efforts dedicated to developing new biodegradable and eco-friendly composites [1,2]. In recent years, bamboo fiber-reinforced polypropylene (PP) composites have played an increasing role in interior automotive parts as well as exterior structural materials due to their flexibility in processing, superior durability, light weight, low cost, biodegradability and recyclability [3,4,5]. In order to extend the application fields of BF-reinforced polymer composites, our research groups have studied the comprehensive properties of bamboo fiber-reinforced polypropylene composites, and explored the feasibility of this kind of material for use in automobile lining parts recently [6,7,8]. However, both BF and PP exhibit combustible characteristics. Especially, the limiting oxygen index (LOI) of PP is about 19%, typical of that for a flammable material. To meet the requirements of the automobile industry, it is necessary to improve the flame retardancy of these composites. Although a large quantity of studies have been conducted on the flame retardancy of PP composites reinforced by lignin, starch, bamboo, wood flour and other fillers [9,10,11,12,13], the data on the flame retardant of PP/BF composites is still limited.

Halogenated flame retardants are extensively used in the production of PP composites due to their remarkable effect. However, this kind of flame retardant tends to release toxic and corrosive gas when it burns, leading to serious environmental problems. Thus, it is urgent to find an effective substitute. The need for halogen-free, low toxicity and less smoke emission as well as high efficiency has driven research in the development of alternative flame retardants [14,15,16]. Intumescent flame retardants, which contain phosphorus and nitrogen, are one of the most important types of halogen-free flame retardants. They have attracted a lot of research attention in PP composites in recent years [17,18]. Generally speaking, these phosphorus- and nitrogen-based flame retardants are mainly effective in the condensed and gas phases. On the one hand, phosphorus-based flame retardants can promote formation of an expanded carbonation layer on the material surface during combustion, which is able to block oxygen and heating transfer, imparting the interior material with good heat insulation performance. On the other hand, nitrogen-based flame retardants, which can produce nitrogen oxides during combustion, will act as an inert diluent in the flame and prohibit flame propagation [19,20,21].

Melamine pyrophosphate (MPP), which contains phosphorus and nitrogen, has been widely used to improve the flame retardant performance of different polymers due to its excellent thermal stability and water resistant characteristics [22,23,24]. Yoshihiko et al. [22] compared the impacts of three flame-retardants, ammonium polyphosphate (APP), melamine polyphosphate (MPP) and aluminum hydroxide, on the burning behavior of wood–plastic composites. It was shown by comparison that wood–plastic composites containing APP exhibited better fire performance than MPP owing to the interaction between APP and wood flour, which can produce a layer of char during combustion. As a result, MPP is often used in combination with other flame retardants. MPP and pentaerythritol (PER) were selected by Pang et al. [23] as intumescent flame retardants to study their flame retardant efficacy on banana fiber-reinforced epoxy composites. Results showed that when flame retardants contents reached 40 wt% and the weight ratio of MPP/PER was 2:1, the composites presented the best combustion performance. Lai et al. [24] reported that triazine-based macromolecules (TBM) exert a synergistic effect on MPP when they were used to improve the flame performance of PP. The limiting oxygen index (LOI) of the PP/MPP/TBM composite reached as high as 31% when the contents of MPP and TBM were 16.7 wt% and 8.3 wt% respectively. The flammability of PP with TBM and MPP can be classified with a UL-94 V-0 rating. Furthermore, the peak heat release rate (PHRR), the total heat release (THR) and the mass loss rate (MLR) measured by cone calorimetry were significantly decreased compared to those of untreated PP.

As a new type of halogen-free flame retardant, aluminum hypophosphite (AP) has also attracted great attention because of its high efficiency and thermal stability. It can exhibit a good flame-retardant effect even at a low addition. This is because the content of phosphorus in AP is very high, which is helpful to produce much more char [25,26,27,28,29]. Since AP can release PH_3_, and PH_3_ can be further oxidized to H_3_PO_4_, which may catalyze the formation of carbon layer, it was successfully used by Yang et al. [25] to prepare flame-retardant polystyrene (PS). AP was also a good choice to synergize other flame retardants. Zhao et al. [28] chose melamine cyanurate (MCA) and AP to prepare flame-retardant polypropylene/wood flour composite, and the results indicated that when 20 wt% AP/MCA at a mass ratio of 5:1 was added into the PP/WF composite, their LOI can increase to 29.5% and the burning rating of the composites reached a V-0 rating according to the UL-94. Zhao et al. [29] further discussed the influence of MPP and AP on the flame retardancy of wood flour reinforced polypropylene composites. When 20 wt% MPP/AP at a mass ratio of 3:1 was loaded into the composites, the LOI value of 29.2%, and the burning rating of a UL-94 V-0 rating were obtained respectively.

However, so far, there are few reports about the possible synergistic effects of MPP and AP in BF reinforced PP composites. Therefore, the properties of BF-reinforced PP composites with different MPP and AP contents were studied systematically in this study. The mechanical properties, thermal stability and combustion properties of BF/PP composites were assessed by tensile and bending tests, LOI and cone calorimeter measurements. The mechanism of flame retardance was studied by observation of the microscopic structures of the composites by scanning electron microscopy (SEM). Additionally, the flame retardant mechanism was probed by examining the macroscopic structures of the residues formed after the cone calorimeter tests using digital photographs.

## 2. Materials and Methods

### 2.1. Materials

Bamboo fiber extracted by using an environmentally friendly solvent-extraction technique [8], was purchased from Fujian Haibosi Chemical Technology Co., Ltd. (Fujian, China). The bamboo fibers were 100–200 μm in diameter and 1–5 cm in length. PP fiber was obtained from Taizhou Hailun Chemical Fiber Co., Ltd. (Guangdong, China). The linear density of PP fiber was 11.11 dtex and the length was 6–8 cm. Both MPP and AP were purchased from Dongguan Jixin Plastic Materials Co., Ltd. (Guangdong, China).

### 2.2. MPP/AP-BF/PP Composites Manufacturing

To prepare the MPP/AP-BF/PP composites, BF was firstly placed in the drying oven at 105 °C (DHG-9075A, Yiheng Instrument Co., Ltd., Shanghai, China), making the moisture content of BF lower than 6%. Then the BF/PP mats were prepared by blending BF and PP fibers with a mass ratio of 1:1 using non-woven air flow paving technology. The flame retardant, consisting of MPP and AP with a constant proportion, was added during the paving process. Then, the mixed system was needle-punched to produce the BF/PP mats. MPP/AP-BF/PP composites were manufactured by hot-press molding under 180 °C for 10 min in a 22 cm × 22 cm × 0.4 cm mold. The mold was further cold pressed at room temperature for 5 min immediately after the hot-pressing. Finally, the panel was taken out of the mold for tests. Table 1 illustrates the formulas of MPP/AP-BF/PP composites.

### 2.3. MPP/AP-BF/PP Composites Characterization

#### 2.3.1. Mechanical Properties

Ultimate tensile strength was determined according to GB/T 1447-2005 [30] with dumbbell-shaped specimens. A minimum of five samples were cut from the panels. The testing was conducted on an INSTRON^®^ universal testing machine (UTM; Model 3365) at a crosshead speed of 10 mm/min.

The flexural strength and modulus of the composites were determined using Tinius Olsen (Model Impact 104) according to GB/T 1447-2005 [30]. The test was completed at room temperature with a loading rate of 10 mm/min. The dimension of the specimens for this test were 100 mm × 10 mm × 4 mm. Five replicates for each condition were made and tested.

#### 2.3.2. Scanning Electron Microscopy (SEM)

In order to assess flame retardant dispersion and their interfacial compatibility with wood, the tensile fracture surfaces of composites were examined by the SEM (JEOL JM-6400, Tokyo, Japan, 5 KV). The gold palladium alloy was used for sputtering deposition on the fracture surface of the sample.

#### 2.3.3. Limited Oxygen Index (LOI)

According to the standard ASTM D2863-17 [31], the LOI of the composite was measured by the JF-3 oxygen index instrument (Jiangning analytical instrument company, Nanjing, China). The dimension of the specimens for this test were 100 mm × 10 mm × 4 mm. Five replicates for each condition were made and tested.

#### 2.3.4. Cone Calorimeter (CONE)

The combustion test was carried out by cone calorimetry according to ASTM E1354-17 [32], in which the heat flux was 50 kW m^−2^. The dimension of the specimens for this test was 100 mm × 10 mm × 4 mm. Three replicates for each condition were made and tested.

## 3. Results and Discussion

### 3.1. Mechanical Properties of BF/PP Composites under Different Mass Fractions of Flame Retardant Addition

#### 3.1.1. Tensile Strength

**Figure 1:**
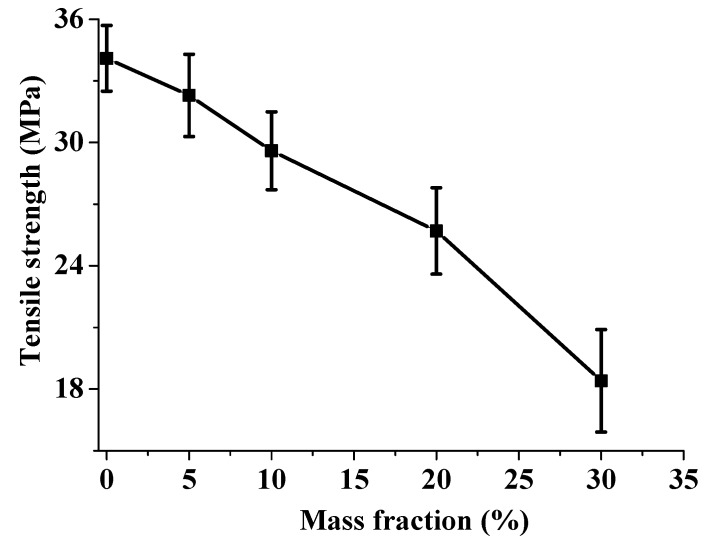
Changes of tensile strength of BF/PP composites with and without flame retardant addition.

Figure 1 presents the tensile strength results with error bars of all composites with and without flame retardant addition. It is evident that the flame retardant has a significant effect on the tensile strength of the composites in which it decreased with the increase of flame retardant mass fraction. This is very noticeable for the composites with 5 wt%, 10 wt%, 20 wt% and 30 wt% flame retardant addition; the tensile strengths decreased by approximately 5.3%, 13.5%, 24.6% and 46.1%, respectively. The comparatively lower tensile properties of BF/PP composites were perhaps a result of the inferior interfacial compatibility between PP and flame retardant. Since MPP and AP are organic and ion salts respectively while PP is a kind of nonpolar polymer. This results in poor compatibility, which promoted the formation of many voids in the composites. Previous research has demonstrated an 11% reduction in the tensile strength of produced chopped E-glass fiber composites due to voids formation [33]. This can be explained by an increase in the concentration of stress in the composites with the increase of the mass fraction of the flame retardant.

#### 3.1.2. Flexural Properties

**Figure 2:**
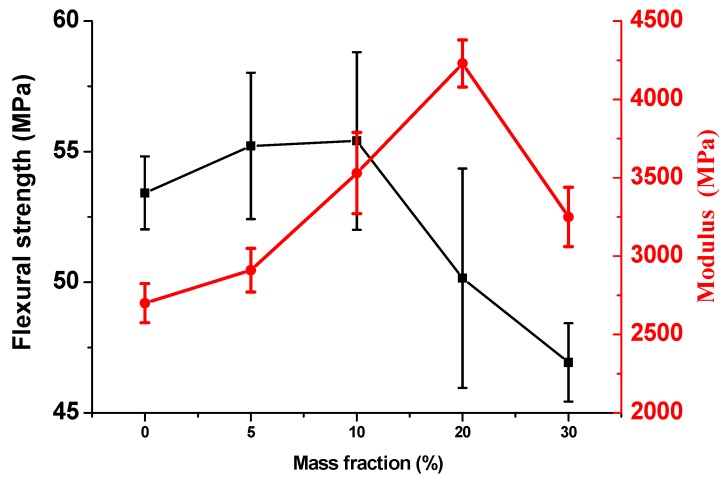
Changes of flexural strength and modulus of BF/PP composites with and without flame retardant addition.

The flexural strength and flexural modulus along with their standard deviations of BF/PP composites are presented in Figure 2. With the increase of flame retardant mass fraction, the strength and modulus exhibited different trends. There is little difference in the flexural strength among untreated BF/PP composite, 5 wt% MPP/AP-BF/PP and 10 wt% MPP/AP-BF/PP composites. However, when the MPP/AP mass fractions exceeded 10 wt%, the flexural strength began to decrease sharply. The BF/PP composites with flame retardant addition presented the highest flexural strength (55.41 MPa) with loading at 10 wt%. Similarly, the flexural modulus also presented substantial improvements of 7.8%, 30.7% and 56.7% with the mass fraction of flame retardant ranging from 0 to 20 wt%, respectively. Although the modulus of the 30 wt% MPP/AP-BF/PP composite decreased, the level was still higher than that of BF/PP composites without the flame retardant. Due to the addition of flame retardant, the stiffness of the resulting composite increased, thus improving the flexural strength and modulus, which was further attributed to the increase of contact interface area [34]. However, when the mass fraction of the flame retardant increased substantially, the excessive flame retardant resulted in inferior interfacial compatibility, and reduced the mechanical properties of the composites.

### 3.2. Micromorphology of BF/PP Composites under Different Mass Fractions of Flame Retardant Addition

**Figure 3:**
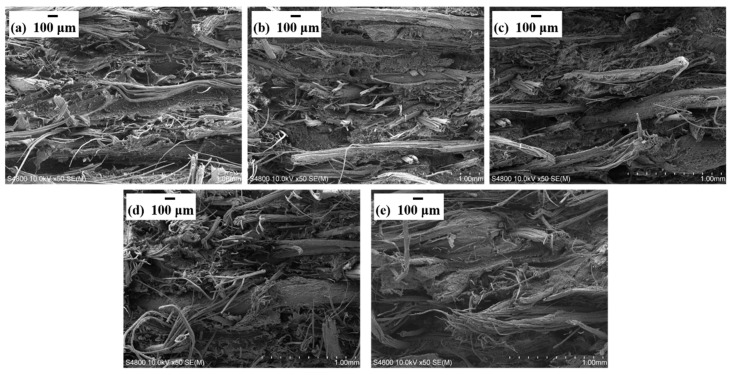
SEM images of fractured surfaces of relevant composites after tensile strength testing: (**a**) BF/PP, (**b**) 5 wt% MPP/AP-BF/PP, (**c**) 10 wt% MPP/AP-BF/PP, (**d**) 20 wt% MPP/AP-BF/PP and (**e**) 30 wt% MPP/AP-BF/PP.

In order to explore the dispersion characteristics of BF and flame retardant in the composite, SEM was used to study the micro morphology of tensile fracture, as shown in Figure 3. It can be observed that BF exhibited a good level of dispersion in the PP matrix by non-woven air flow paving technology. According to Figure 3, the images show similar patterns at low magnification (50×), indicating that the flame retardant had no obvious influences on the dispersion of BF. Moreover, the composites both with and without flame retardant addition exhibited similar fracture characteristics, i.e., the fracture modes of the composites included fiber fracture and pull-out. Therefore, it can be preliminarily inferred that the change in mechanical properties of the composite has no significant relationship with the distribution of fibers, but may be related to the addition of the flame retardant.

**Figure 4:**
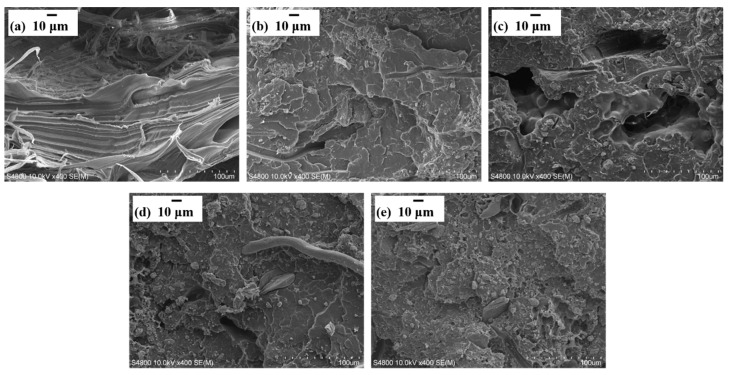
SEM images of the distribution of flame retardant in the relevant composites after tensile strength testing: (**a**) BF/PP, (**b**) 5 wt% MPP/AP-BF/PP, (**c**) 10 wt% MPP/AP-BF/PP, (**d**) 20 wt% MPP/AP-BF/PP and (**e**) 30 wt% MPP/AP-BF/PP.

To confirm this hypothesis, the micro-morphologies of the composites were characterized at higher magnification (400×), the results of which are shown in Figure 4. It can be seen that the flame retardant was distributed uniformly in the PP matrix. As presented in Figure 4a, the fractured surfaces of the composites without flame retardant addition were smooth. However, with the increase of the flame retardant content, the fracture surfaces presented increasing roughness (Figure 4a–e). This is because these flame retardants caused a significant amount of stress concentration. In Figure 4e in particular, many small holes were created on the surfaces, which confirms the poor compatibility between flame retardant and PP matrix, and further explains the deterioration of the composites mechanical strength.

### 3.3. LOI Results of BF/PP Composites under Different Mass Fractions of Flame Retardant Addition

**Figure 5:**
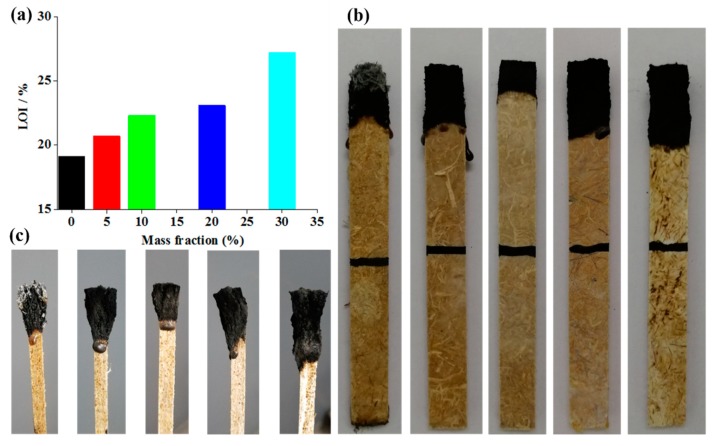
The limiting oxygen index (LOI) test and the char layers morphology of BF/PP composites after the LOI test with and without flame retardant addition: (**a**) LOI value, (**b**) char layer morphologies on sample front, (**c**) char layer morphologies on sample side.

Flame retardancy of the BF/PP composite was investigated by LOI testing. Figure 5 summarizes the main results of BF/PP composites with and without flame retardants. The LOI value of the BF/PP composite was 19.1% (Figure 5a) and hence highly flammable. LOI values of the BF/PP composites increased continuously with the increase of the flame retardant mass fraction. Values for the 5 wt% MPP/AP-BF/PP, 10 wt% MPP/AP-BF/PP, 20 wt% MPP/AP-BF/PP and 30 wt% MPP/AP-BF/PP composites were 20.7%, 22.3%, 23.1% and 27.2%, respectively, which means the synergistic effect of MPP and AP could effectively improve the flame retardancy of BF/PP composites.

After the LOI test, the char layer morphologies on both of the sample front and side were carefully analyzed, and the results are shown in Figure 5b,c, respectively. As can be seen from the figure, the residue of the BF/PP composite without flame retardant addition was less intense and loosely structured, and the char was composed of only some ash, and could easily collapse and fall down. In addition, the composite was prone to flame during combustion. However, when the flame retardant was introduced into the composites, the samples presented a char residue formed on the surfaces after burning. This kind of char layer can isolate oxygen and heat by covering the surface of the composites, and the flame dripping disappeared gradually with the increasing of flame retardant addition.

### 3.4. Flame Retardancy of BF/PP Composites under Different Mass Fractions of Flame Retardant Addition

In order to investigate the influence of MPP/AP addition on the flame retardancy of the BF/PP composites, cone calorimetry was carried out in this study to measure the time to ignition (TTI), peak heat release rate (p-HRR), total heat release (THR), total smoke production (TSP) and mass loss values for the BF/PP composites. Table 2 lists the data with the addition of MPP/AP from 0–30 wt%.

#### 3.4.1. TTI

TTI is an important parameter to determine a flame-retardant’s effectiveness on ignitability. The TTI of BF/PP was only 16 s as seen in Table 2. However, after adding MPP/AP flame retardant, it began to increase. When 30 wt% of the MPP/AP was loaded, TTI valued increased to 24 s, indicating that MPP and AP have a good ability to slow down the combustion of BF/PP composites.

#### 3.4.2. HRR and THR

**Figure 6:**
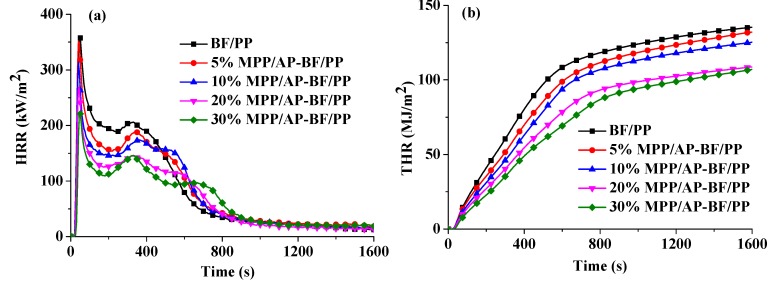
Heat release rate (HRR; **a**) and total heat release (THR; **b**) curves of BF/PP composites with and without flame retardant addition.

Figure 6a,b, which illustrates the HRR and THR values as a function of time respectively, shows the combustion behaviors of the composites. The BF/PP composite without flame retardant addition burned rapidly after ignition. As shown in Figure 6a, it had the highest peak heat release rate of 360 kW/m^2^. In contrast, the p-HRR values of the BF/PP composites with 5 wt%, 10 wt%, 20 wt% and 30 wt% flame retardant addition reduced to 351, 314, 269 and 221 kW/m^2^, respectively. The HRR curve of the BF/PP composite without flame retardant addition comprised of two stages. The first stage was the combustion of gases generating from the thermal decomposition of composites ignited by the high-voltage arc. The second stage was primarily owing to the pyrolysis and combustion of cellulose at higher temperature. However, the BF/PP composites with flame retardant addition exhibited different curve patterns with three stages. The last stage may be caused by the flameless combustion of the char layer. Moreover, the THR value of the BF/PP composite was 135 MJ/m^2^. With the addition of MPP/AP mass fraction, the THR value of composites decreased rapidly. As shown in Figure 6b, the THR value of the BF/PP composites with 5 wt%, 10 wt%, 20 wt% and 30 wt% flame retardant addition decreased by 2.4%, 7.5%, 19.7% and 20.9%, respectively compared with the untreated BF/PP composites. These two results demonstrated the effectiveness of MPP/AP present in BF/PP composites.

#### 3.4.3. TSP Analysis

**Figure 7:**
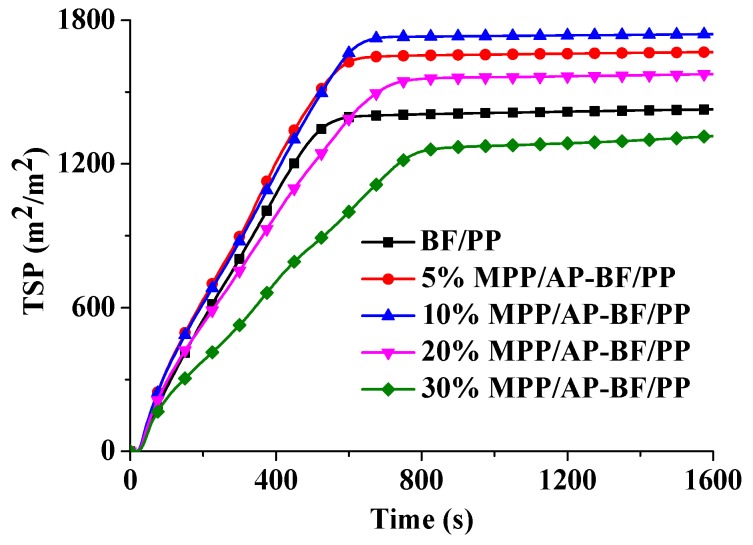
Total smoke production (TSP) curves of BF/PP composites with and without flame retardant addition.

Figure 7 presents the TSP values of the BF/PP composites with different MPP/AP addition. The TSP of BF/PP composites without flame retardant was 1427 m^2^/m^2^. With the increasing of the flame retardant mass fraction, TSP of the composites first increased slightly, and then decreased significantly after the mass fraction exceeded 10 wt%. The 10 wt% MPP/AP-BF/PP composite had the highest TSP (1742 m^2^/m^2^). This is because many nonflammable gas products (flammable and non-flammable gas) were generated because of the addition of MPP/AP. Additionally, the MPP/AP may have the ability to prohibit the flammable gases from burning, which may also result in the higher TSP value. However, with the further increase of the mass fraction, the TSP reduced obviously. This is because excessive flame retardant could not only inhibit the flammable gases from burning, but also inhibit the decomposition of composites. It could be inferred that excessive MPP and AP played a very good suppression role in smoke production.

#### 3.4.4. Flame Retardant Mechanism of MPP/AP

**Figure 8:**
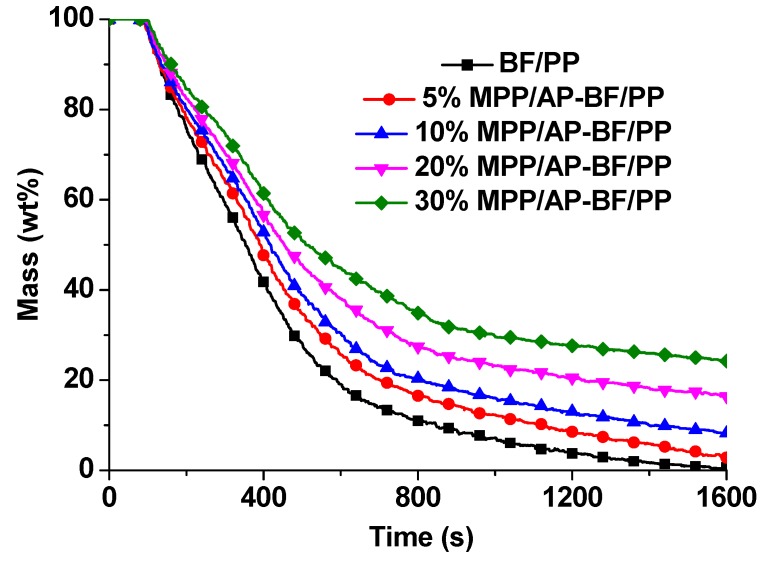
Mass loss of BF/PP composites with and without flame retardant addition.

**Figure 9:**
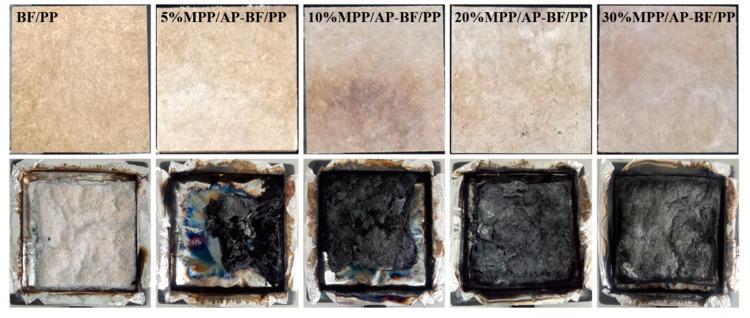
Surface topography of BF/PP composites with and without flame retardant addition before and after cone calorimetric testing.

In order to clarify the flame retardant mechanism of MPP and AP in BF/PP composites, the mass loss values and photographs of BF/PP composites before and after cone calorimetric exposure were characterized as shown in Figure 8 and Figure 9, respectively.

As exhibited in Figure 8, the mass loss rate gradually decreased with the increase of flame retardant mass fraction. For the BF/PP without flame retardant addition, the mass loss at 1600 s was 0.7%, which indicates that the composites were burned completely. This conclusion can be supported by the photographs in Figure 9. It can be observed that there was a small amount of white residue left in the aluminum tray. However, when the flame retardant was added into the BF/PP composites, increasingly much more char residue remained after the cone calorimetric exposure, which is verified by the data in Table 2 and Figure 9. In addition, the formation of char residue appeared to be more compact, and the results were consistent with the mass loss (Figure 8). Overall, the MPP and AP presented as flame retardants had a positive effect on BF/PP composites.

**Figure 10:**
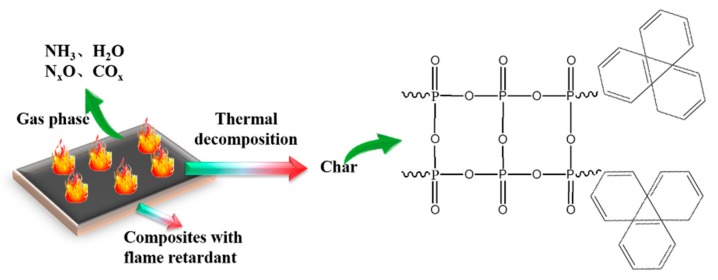
Flame retardant mechanism of MPP/AP.

Based on the literature, a possible series of mechanistic stages may be proposed. At high temperatures, MPP decomposed into some non-flammable gas products (including NH_3_, NO and H_2_O), as well as some phosphorus-containing substances [22,23,24]. Additionally, AP can also decompose at high temperatures to release Al_2_(HPO_4_)_3_ and PH_3_ [28,29]. These gases can not only dilute flammable gases, but also act as a gas shield on the surfaces of materials, thus reducing combustion. In addition, these two flame retardants can volatilize phosphorus–oxygen reactive free radicals into the gas, capturing the free •OH and •H to terminate the main chain scission of PP. Finally, the phosphorus substances from the composition of MPP and AP could react with the –OH groups in BFs to form a P–O–C cross-linking layer and promote the formation of carbon layer on the surface of the BF/PP composite (Figure 10). Thus it may be proposed that MPP and AP can simultaneously exhibit condensed-phase and gas-phase flame retardant effects in BF/PP composites to enhance their flame retardancy.

## 4. Conclusions

In this paper, the effects of MPP/AP at a constant mass ratio of 2:1 on the mechanical properties, morphology and flame retardancy of BF reinforced PP composites were studied, and the coordinated flame retardant mechanism of MPP/AP was discussed. The results showed that the addition of MPP and AP had different effects on the tensile and bending properties of the composite. With the increase of the mass fraction of MPP/AP, the tensile strength decreased gradually, while the flexural strength and modulus first slowly increased, and then quickly decreased. The highest flexural strength and modulus of the flame retardant composites were 55.4 MPa and 4230 MPa, respectively, exhibiting considerable improvements of 3.72% and 56.7% compared with the BF/PP composite without flame retardant addition. Through LOI and cone calorimetric testing, it was determined that the composites exhibited better flame retardant characteristics with the addition of MPP and AP. Moreover, compared with the BF/PP composites without flame retardant, the increased addition of MPP/AP content into BF/PP composites could result in the increase of LOI, and the decrease of p-HRR and THR values by 38.5% and 20.9%, respectively. In addition, MPP and AP could play the role in both condensed and gas phase in the combustion process. All the results indicate that the combined use of MPP and AP at a ratio of 2:1 had a satisfactory flame retardant effect on BF/PP composites.

## Figures and Tables

**Table 1:** The formulas of MPP/AP-BF/PP composites.

Sample	BF (wt%)	PP (wt%)	MPP (wt%)	AP (wt%)	Mass Ratio (MPP:AP)
BF/PP	50	50	0	0	0:0
5 wt%MPP/AP-BF/PP	47.5	47.5	3.33	1.67	2:1
10 wt%MPP/AP-BF/PP	45	45	6.67	3.33	2:1
20 wt%MPP/AP-BF/PP	40	40	13.33	6.67	2:1
30 wt%MPP/AP-BF/PP	35	35	20	10	2:1

**Table 2:** Cone calorimetric analysis results of BF/PP composites with and without flame retardant addition.

Sample	BF/PP	5% MPP/AP-BF/PP	10% MPP/AP-BF/PP	20% MPP/AP-BF/PP	30% MPP/AP-BF/PP
TTI (s)	16	18	19	21	24
p-HRR (kW/m^2^)	360	351	314	269	221
THR (MJ/m^2^)	135	132	125	109	107
TSP (m^2^/m^2^)	1427	1667	1742	1575	1316
Char remains at 1600 s (%)	0.7	2.8	8.3	16.4	24.3
